# The role of biopsychosocial factors in classifying pain intensity across various chronic pain conditions

**DOI:** 10.1038/s41598-026-46612-9

**Published:** 2026-05-29

**Authors:** Iara De Schoenmacker, Maria Monzon, Laura Sirucek, Paulina S. Scheuren, Robin Lütolf, Lindsay M. Gorrell, Florian Brunner, Armin Curt, Jan Rosner, Petra Schweinhardt, Michèle Hubli, Catherine R. Jutzeler

**Affiliations:** 1 Biomedical Data Science Lab, Department of Health Sciences and Technology (D-HEST), ETH Zurich, 8092 Zurich, Switzerland; 2 Spinal Cord Injury Center, Balgrist University Hospital, University of Zurich, Zurich, Switzerland; 3 Neuroscience Center Zurich, University of Zurich, Zurich, Switzerland; 4 Department of Chiropractic Medicine, Integrative Spinal Research Group, Balgrist University Hospital, University of Zurich, Zurich, Switzerland; 5 Center for Neuroplasticity and Pain (CNAP), Department of Health Science and Technology, Aalborg University, Aalborg, Denmark; 6 International Collaboration on Repair Discoveries, University of British Columbia, Vancouver, BC Canada; 7 Department of Anesthesiology, Pharmacology and Therapeutics, Faculty of Medicine, University of British Columbia, Vancouver, BC Canada; 8 Physical Medicine and Rheumatology, Balgrist University Hospital, Zurich, Switzerland; 9 Danish Pain Research Center, Department of Clinical Medicine, Aarhus University, Aarhus, Denmark; 10 Swiss Institute of Bioinformatics (SIB), Lausanne, Switzerland; 11 Biomedical Data Science Lab, Swiss Federal Institute of Technology (ETH), Lengghalde 2, 8008 Zurich, Switzerland

**Keywords:** Chronic pain, Pain classification, Biopsychosocial model, Quantitative sensory testing, Machine learning, Neuroscience, Sensory processing

## Abstract

Chronic pain is a multifaceted condition shaped by biological, psychological, and social factors, which challenges its diagnosis and treatment. While prior research largely focused on sensory profiles to distinguish pain mechanisms, this study adopted a biopsychosocial perspective with the key addition of experimental pain paradigms to identify factors explaining variance in chronic pain severity across diverse etiologies. The dataset comprised 101 individuals with chronic pain and 63 pain-free controls. Participants were classified into three groups (no pain, mild-to-moderate pain, and severe pain) using three models: a sensory-profile-based model including quantitative sensory testing (QST model), a biopsychosocial model incorporating QST alongside psychological and social variables (biopsychosocial model), and a model using the same biopsychosocial data but excluding QST measures (noQST model). The QST model achieved an accuracy of 0.51 and an F1-score of 0.48. In contrast, the biopsychosocial model performed best, with both accuracy and F1-score of 0.71, while the noQST model reached 0.60 for both metrics. Key predictors in the best-performing model included quality of life, loss of sensation, depressive mood, pain catastrophizing, anxiety, fatigue, and general health. Overall, biopsychosocial factors enhanced chronic pain severity classification beyond sensory profiles alone across heterogeneous pain etiologies.

**Supplementary Information:**

The online version contains supplementary material available at 10.1038/s41598-026-46612-9.

## Introduction

Chronic pain remains a complex and poorly understood condition, presenting significant challenges for both diagnosis and treatment. A major obstacle is identifying the underlying mechanisms that drive chronic pain, which often vary significantly between individuals. Previous studies often but not exclusively sought to identify subgroups of common underlying pathomechanisms inferred from sensory profiles^1–8^. Similarly, our group previously aimed at clustering individuals with different chronic pain conditions, i.e., complex regional pain syndrome (CRPS) and low back pain (LBP), based on their quantitative sensory testing (QST) profile assessed in the most painful area^9^. This approach strived to identify sensory patterns that might suggest distinct pathomechanisms as well as disclosing the origin of such pain-generating mechanisms, e.g., peripheral, spinal, or supra-spinal^10^, as proposed previously^11^ regardless of the clinical diagnosis. While two distinct subgroups emerged with potentially different underlying pathomechanisms^9^, many individuals with LBP exhibited sensory profiles similar to those of pain-free controls, raising questions about whether QST alone is suitable to detect clinically relevant clusters. Adding psychological and social factors as well as complementary experimental paradigms might improve clustering performance.

The biopsychosocial model of pain has gained traction in the understanding and treatment of chronic pain, recognizing that psychological and social factors, in addition to biological ones, play crucial roles in shaping pain perception^12^. Recent work using data from this study^13^ explored how psychological and physiological factors contribute to acute pain perception. However, their focus was on acute, experimentally-induced, rather than chronic, spontaneous pain. Another study developed a model that predicted the incidence, severity, and spread of chronic pain based on biopsychosocial factors in individuals with various painful and non-painful medical conditions^14^. Factors like fatigue, stress, and depression predicted the spread of chronic pain. However, the model did not include experimental paradigms indicative of nociceptive processing (i.e., biological factors). Comprehensive pain paradigms can illuminate the balance between anti- and pronociceptive processes relevant for chronic pain^15^. Various approaches, including the investigation of the spinothalamic integrity^16^, temporal summation of pain (TSP)^17,18^, conditioned pain modulation (CPM)^18,19^, and experimental pain habituation^20,21^ offer valuable insights into peripheral, spinal, and supra-spinal sensitization, as well as top-down modulation processes^11^.

We aimed to classify individuals with differing chronic pain intensity using three classification models: one that focused solely on sensory profiles (QST model), one integrating a broad set of biological, psychological, and social features (biopsychosocial model), and one including the same data as the latter model but excluding the QST-data (noQST model). The biopsychosocial model incorporated experimental pain paradigms such as TSP, CPM and experimental pain habituation, alongside psychological variables (depression, anxiety, pain catastrophizing) and broader health-related factors such as quality of life, fatigue, and general health. The study population consisted of a diverse chronic pain cohort, including individuals with CRPS, LBP, and neuropathic pain after spinal cord injury (SCI), and pain-free healthy controls (HC). This heterogeneous sample was intended to enhance the study’s ability to identify the most common and influential factors contributing to the perceived severity of chronic pain independent of its etiology. We hypothesized that the biopsychosocial model would significantly outperform the QST model and noQST model in classifying individuals with differing perceived chronic pain intensities. Additionally, and most importantly, we examined the feature importance in all models which potentially infer the underlying mechanism of why some patients experience severe pain regardless of their clinical diagnosis. For the biopsychosocial model we hypothesized that, in addition to factors related to pain perception and modulation, psychological aspects such as pain catastrophizing would play a pivotal role.

## Methods

### Study population

This study is a secondary analysis of a previously acquired large dataset recruited for the Clinical Research Priority Program (CRPP) Pain funded by the University of Zurich, Switzerland. Participants with chronic pain and age- and sex-matched healthy controls (HC), aged 18 to 80 years, were recruited between November 2019 and April 2022 via the Balgrist University Hospital in Zurich, Switzerland. Participants with CRPS were enrolled via the Department of Rheumatology, participants with LBP through the Department of Chiropractic Medicine, and participants with neuropathic pain after SCI from the Spinal Cord Injury Center. Additionally, some participants with LBP were recruited through advertisements in Swiss chiropractic clinics and medical journals. Exclusion criteria included pain duration of less than three months, self-reported neurological conditions (except SCI, such as polyneuropathy or radiculopathy), systemic diseases (e.g., autoimmune disorders, diabetes), psychiatric conditions, pregnancy, or inability to comply with study instructions. Participants with SCI were excluded if their injury occurred less than one year ago or if the neurological level of injury was above C8, ensuring intact upper limb sensorimotor function. Participants with LBP were excluded if their pain was related to serious underlying pathology, such as infections, fractures, or inflammation. Healthy controls had the same exclusion criteria as participants with chronic pain, with the additional requirement of no acute pain, no history of chronic pain lasting more than three months, and no LBP episodes longer than three consecutive days within the past year. Moreover, due to time constraints of the study, not all pain patients could be matched with a healthy control; therefore, matching was limited to a subset of the patient sample. Healthy controls were selected based on the best available match for age and sex.

Prior to the study, all participants (patients and HC) underwent sensory testing in a non-painful area (e.g., hand or shoulder) using bedside assessments for vibration, thermosensation, pinprick, and light touch. If a participant displayed a noticeable sensory deficit, the findings were reviewed with a neurologist (JR), and the participant was excluded from the study if necessary. Written informed consent was obtained from all participants, and the study was conducted in accordance with the Declaration of Helsinki. Ethical approval was granted by the Cantonal Ethic Commission Zurich (original: ‘Kantonale Ethikkommission Zürich, KEK’) (EK-04/2006, PB_2016-02051, PB_2019-00136) and the study was registered on clinicaltrials.gov (NCT02138344 and NCT04433299).

### Study protocol

The experimental protocol of the overarching project (CRPP Pain) consisted of a comprehensive testing battery (2 visits of 3 h each), including psychophysical pain assessments, electrophysiological measures, and evaluation of psychological and social factors potentially contributing to the experience of chronic pain. A detailed description of the included data can be found in the following sections.

#### Biopsychosocial factors

##### Questionnaires

Various questionnaires were administered to assess biopsychosocial factors potentially contributing to the perceived chronic pain intensity. Participants completed these electronically through a link provided outside of the two in-person visits. The questionnaires included the following:

- assessment of age, sex, general health, and fatigue
- the International Physical Activity Questionnaire—Short Form (IPAQ-SF^22^)
- the Multidimensional Assessment of Interoceptive Awareness (MAIA^23^)
- assessment of the relationship status, education, and quality of life
- the Pain Catastrophizing Scale (PCS^24^)
- the Hospital Anxiety and Depression Scale (HADS^25^)

All questionnaires were completed in German. General health was assessed with the question, “How would you assess your general health?” (original: Wie schätzen Sie Ihre allgemeine Gesundheit ein?), with response options ranging from excellent, very good, good, less good, to bad (original options: ausgezeichnet, sehr gut, gut, weniger gut, schlecht). Fatigue was assessed by asking, “How much have you suffered being tired in the past week?” (original: Wie stark haben Sie in der letzten Woche unter Müdigkeit gelitten?), with the answer options of no problems, slight problems, moderate problems, and severe problems (original options: keine Probleme, leichte Probleme, mässige Probleme, and schwere Probleme). In addition to these questionnaires, psychophysical and electrophysiological assessments were performed. Furthermore, participants could select their relationship status from the following options: single, in a committed relationship/married, separated/divorced, or widowed (original options: ledig, feste Partnerschaft/verheiratet, getrennt lebend/geschieden, and verwitwet). Educational background was categorized into the options: compulsory schooling, apprenticeship, secondary school, higher technical college, university of applied sciences, and university (original options: obligatorische Schulzeit, Berufslehre, Mittelschule, höhere Fachhochschule, Fachhochschule, and Universität). Quality of life was evaluated through the question, “When you look back at the past week, how would you rate your quality of life?” (original: Wenn Sie auf die letzte Woche zurückblicken, wie beurteilen Sie Ihre Lebensqualität?), with response options being very good, good, moderate, bad, and very bad (original options: sehr gut, gut, mittelmässig, schlecht, and sehr schlecht).

##### Psychophysical assessments

Psychophysical assessments included QST, TSP, CPM, and experimental pain habituation. For each participant, the psychophysical assessments were performed in two different body areas: the area with the highest reported pain intensity (i.e., the most painful area) and a remote, pain-free body area (i.e., control area). Typically, the control area was the non-dominant hand. For participants with CRPS in whom the contralateral hand was affected, the contralateral shoulder was used as a remote, pain-free body area. This approach was based on prior findings in CRPS that pain symptoms can spread to the opposite limb^26^. Age- and sex-matched healthy controls (HC) were assessed in the same body areas as the corresponding participants with chronic pain. These psychophysical assessments are briefly outlined in the following: for detailed descriptions please refer to previous publications from this project (QST:^9^, TSP:^17^, CPM:^18^, experimental pain habituation:^27^).

##### Quantitative sensory testing (QST)

QST was conducted during the initial visit, adhering to the guidelines by the German Research Network on Neuropathic Pain (DFNS)^28^ and conducted by trained experimenters. The complete QST protocol was conducted in the most painful area, while the control area focused on assessing widespread hyperalgesia rather than sensory loss due to time constraints. Thus, only QST measures evaluating sensory gain of function were performed in the control area, including cold pain threshold (CPT), heat pain threshold (HPT), mechanical pain threshold (MPT), stimulus–response function (SR-function), pressure pain threshold (PPT), and wind-up ratio (WUR). For the SR-function, only two applications per stimulus intensity were applied in the control area, compared to five in the painful area. In line with the DFNS protocol, the control area was always assessed first. QST data were z-transformed for each participant using eQuiSTA software (version 1.3.4) provided by the DFNS. The transformed scores were capped at a maximum and minimum Z-score of ± 4 standard deviations, respectively. The cut-off of 4 standard deviations was chosen, because according to the Z-distribution, approximately 99.99% of the data lies within this interval.

##### Temporal summation of pain (TSP)

In addition to the WUR of the QST protocol we included a more detailed assessment of TSP. This assessment was always performed in the second visit and was measured using twelve consecutive pinprick stimuli (MRC Systems, Heidelberg, Germany) applied at a frequency of 0.33 Hz. The order of the testing area was randomized. The stimulation intensity was individually adapted for each participant, targeting a level of 4 on a numeric rating scale (NRS) from 0 to 10 (0: no pain, 10: most intense pain tolerable). This target level was chosen to establish a solid baseline pain intensity while minimizing the risk of an early ceiling effect. The NRS 4 was determined using a staircase method immediately before the TSP procedure, with a minimum and maximum intensity of 8 mN and 512 mN, respectively.

Throughout the TSP protocol, participants rated each stimulation on the NRS. To determine the magnitude of TSP, the average pain rating from the first three stimulations (baseline) was subtracted from the average rating of the last three stimulations. The TSP magnitude was then normalized to the baseline.

While we also assessed TSP using thermal (hot) and other mechanical stimuli (pressure), we chose to adhere to the most frequently used paradigm (i.e., pinprick stimulation) for this study.

##### Conditioned pain modulation (CPM)

CPM was always evaluated after the TSP assessment in the second visit. The CPM protocol was conducted twice, once with the test stimulus (TS) being applied in the control area and once in the most painful area, in a randomized order with a 10-min break between the two protocols. The TS involved measuring the PPT (Pressure Algometer FDN 100, Wagner Instruments, USA) within the corresponding testing area. The conditioning stimulus (CS) involved immersing either the contralateral hand or foot of the control area into a cold water bath set at 9 ± 0.5 °C. The water temperature was continuously monitored using a digital bottle thermometer (Reer, Germany). Water circulation was maintained by a magnetic stirrer (IKA® RET basic C, Huber & Co. AG, Switzerland). The CS was applied for a duration of two minutes. Participants rated their perceived pain due to the CS using the NRS at three time points: upon insertion, 30 s after immersion, and at the end of the 2-min immersion.

The TS was assessed once before the CS and again 30 s after CS initialization. The CPM effect was calculated as the relative change in PPT compared to the baseline measurement, using the following equation (Eq. 1):

1 $$CPM\,effect=\frac{({Pre}_{PPT}-{During}_{PPT})}{{Pre}_{PPT}}*100$$

Similar to the TSP protocol, the CPM protocol was also performed using another CS (lukewarm water) and TS (HPT). Additionally, a sequential paradigm has been performed. However, for this study, we adhered to the most frequently used paradigm that also showed the most consistent CPM effect.

##### Subjective experimental pain habituation

Experimental pain habituation in response to noxious contact-heat stimuli was randomly assessed at the start of either the first or second visit. Two blocks of 16 to 20 noxious contact-heat stimuli were delivered using a 27mm diameter thermode (CHEP thermode, PATHWAY Pain and Sensory Evaluation System; Medoc, Ramat Yishai, Israel). The number of stimuli per block was determined by the investigator based on live monitoring for blink artifacts and alpha wave contamination (based on the electroencephalography (EEG) recordings described in Section “Electrophysiological assessments”), aiming to achieve at least 16 artifact-free trials. The thermode allowed for a rapid heating rate of 70 °C/s and a cooling rate of 40 °C/s. The target temperature for the noxious heat was set at 52 °C, starting from a baseline of 42 °C. If participants found the stimuli intolerable during the familiarization phase, the protocol was adjusted by lowering the baseline temperature to 35 °C, while maintaining the target temperature at 52 °C^29,30^. The inter-stimulus-interval was randomized between 13 and 17 s, with a 2 to 3-min break between the two stimulation blocks. To minimize peripheral adaptation and receptor fatigue, the thermode was slightly repositioned within the tested area after each stimulus^31^.

After each stimulus, participants rated their perceived pain using the NRS. To assess experimental pain habituation, the relative change in the average pain rating between the first and second stimulation block was calculated (Eq. 2) both containing the same number of stimuli.

2 $$Experimental\,pain\,habituation= \frac{({Block}_{2}-{Block}_{1})}{{Block}_{1}}*100$$

Experimental pain habituation was also assessed using pinprick stimuli. However, as for the other experimental pain paradigms, we adhered to the most frequently used stimulation modality.

##### Electrophysiological assessments

In addition to recording pain ratings following contact-heat stimuli as described in Section “Experimental pain habituation”, also contact-heat evoked potentials (CHEPs), and pain-induced sympathetic skin responses (SSRs) were recorded. The setup for the CHEPs (i.e., EEG) and SSR recordings is described elsewhere^27^.

The recording of CHEPs and SSRs enabled the investigation of experimental pain habituation in a more objective manner. For CHEP habituation, the relative change in CHEP amplitude between the first and second stimulation block was calculated (Eq. 2). For SSR habituation, a within-block comparison of the first stimulation block was conducted, since a pronounced SSR habituation is already observable after the first few stimuli^32^. The relative change was assessed by comparing the average response of the first three stimuli (1, 2, and 3) to the average response of stimuli 8, 9, and 10.

In order to evaluate the integrity of the spinothalamic tract, CHEPs were classified as either normal or abnormal. A CHEP was considered abnormal if the peak N2 latency exceeded the expected value by more than two standard deviations of normative data^29,33^ or if no response could be recorded. Otherwise, the CHEP was classified as normal.

The integrity of the dorsal columns was evaluated using somatosensory evoked potentials (SSEPs), recorded with the Keypoint G4 Workstation (Dantec Medical, UK) and a hand-held stimulation electrode (9013L0222, Natus Neurology Incorporated, USA). The stimulation site for SSEPs was selected based on the participant’s most painful area. If the most painful area was in the upper limbs, stimulation was applied to the median nerve of the affected limb. For painful areas in the back or lower limbs, the tibial nerve was stimulated. In case of SCI, the dermatome corresponding to the most painful area was stimulated using dermatomal SSEPs. Stimulation for dermatomal SSEPs was delivered via two disposable disk electrodes (019-400400, Natus Neurology Incorporated, USA). A total of 300 electrical stimuli were applied, with a stimulation frequency of 3.1 Hz and a pulse duration of 0.2 ms. The corresponding SSEPs were recorded using two disposable needle electrodes (MN3512P150, Spes Medica). Electrode placement followed the 10–20 international system, with C3’ or C4’ used for upper limb stimulation and Cz’ for lower limb stimulation, while the reference electrode was placed at Fz. A wet wristband was used as the ground electrode. The sampling rate was 24,000 Hz, and recordings were band-pass filtered between 2 and 2000 Hz. The 300 recorded SSEPs were averaged offline. Two independent investigators evaluated the SSEPs for the presence of N- and P-peak latencies within expected time windows, based on established normative values^34,35^. The SSEPs were then classified as either normal or abnormal. An SSEP was considered abnormal if the peak latency exceeded the expected value by more than two standard deviations or if no response could be recorded. Otherwise, the SSEP was classified as normal^24,25^.

### Classification model

Three classification models were compared: one that included only the sensory profile of the most painful area (QST model), one incorporating the same sensory features along with additional biopsychosocial features (biopsychosocial model), and one including the same data as the latter model, but excluding the sensory profile of the most painful area (noQST model). The classification target label was based on participants’ self-reported maximal pain intensity over the preceding four weeks, measured on a 0–10 NRS, and categorized into three distinct levels. The maximal pain intensity was chosen because recalling a maximal pain intensity is potentially easier and more accurate than recalling an average pain intensity. The pain intensity classes were defined as: no pain (NRS 0, i.e., HC), participants with mild-to-moderate pain (NRS 1–6), and participants with severe pain (NRS 7–10)^36^. These classes were chosen in order to have a more balanced label (similar number of participants for each label) for the classification model.

Given the relatively small dataset, features with more than 10% missing values were excluded from the analysis^37^. The percent missingness for each feature can be found in the Supplementary Tables S1 and S2. Additionally, since the information regarding the participant cohort inherently identifies participants with no pain (i.e., HC), this feature was also removed.

**Fig. 1:**
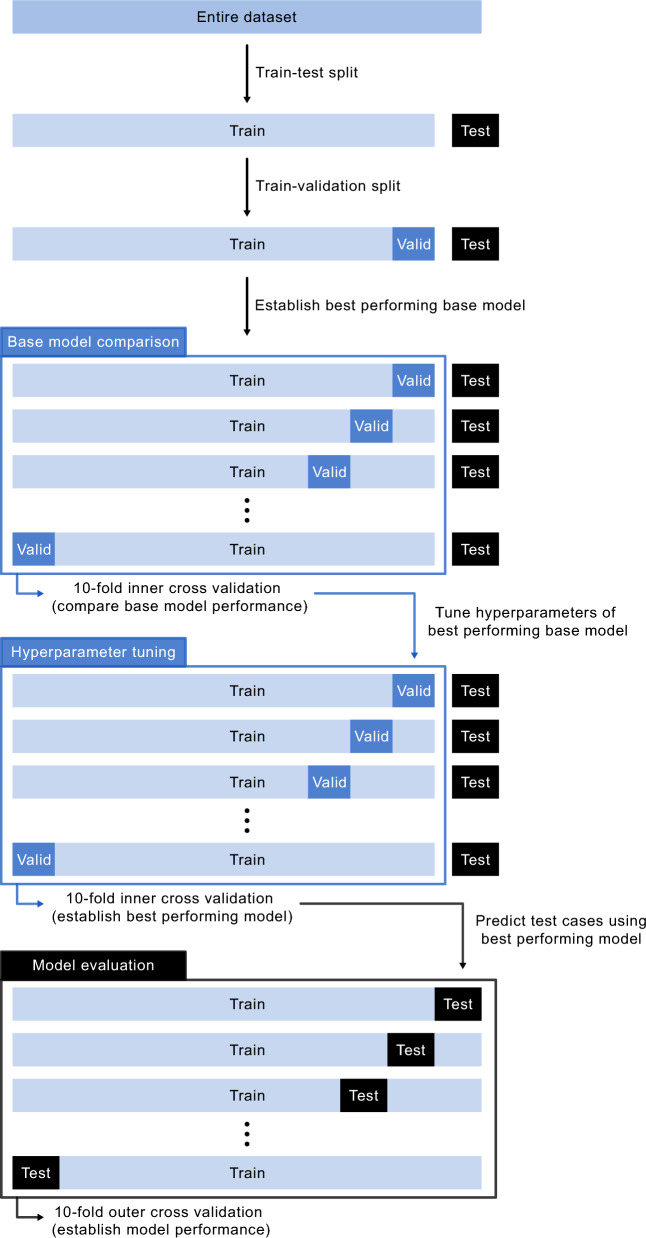
Classification model pipeline. The dataset is split into train, validation (valid) and test set. The validation set is used to establish the best performing base model as well as for hyperparameter tuning. The test set is used to establish the final classification model performance.

The classification model was developed using PyCharm (version 2022.3.3; https://www.jetbrains.com/pycharm/) with the PyCaret library (version 3.0; https://pycaret.gitbook.io/docs) in Python (version 3.10; https://www.python.org/). To mitigate the risk of overfitting due to the limited sample size, we implemented a nested cross-validation approach with a tenfold outer loop to evaluate model performance and a tenfold inner loop to identify the best performing base model and hyperparameter tuning (Fig. 1). Importantly, a stratified outer train-test split based on the target label was performed prior to data imputation and hyperparameter tuning to prevent information leakage between the train and test sets.

Numerical features with missing values were imputed using five iterations of Random Forest imputation. These variables were then normalized using min–max scaling (i.e., 0 to 1) to ensure uniform feature scales. Categorical or ordinal variables with missing values were imputed using five iterations of Light Gradient-Boosting Machine imputation. These variables were one-hot encoded (transforming categorical values into binary vectors with 1 and 0) for further analysis.

Feature selection was conducted using the *SelectKBest* method from the *scikit-learn* library. To mitigate potential multicollinearity and reduce dimensionality, features with a statistical variance below 0.01 and those highly correlated (with a Pearson correlation coefficient of 0.7) were removed to reduce the risk of overfitting. Three feature sets were created: one based solely on the sensory profile (QST) of the most painful area, one that also included all additional biopsychosocial features, and one with all the additional biopsychosocial feature excluding the sensory profile of the most painful area (noQST). To reduce the dimensionality and ensure comparability, 70% of QST features, 20% of biopsychosocial features, and 30% of the noQST features were selected, resulting in nine features each. More specifically, the threshold for the biopsychosocial dataset was set at 20% to minimize the risk of overfitting due to the limited sample size. Subsequently, the threshold for the QST and noQST dataset was adjusted to 70% and 30% respectively to achieve an equivalent number of features. By training all models on an equal number of features, we aimed to ensure that differences in performance reflected the informational content of the feature domains rather than differences in model dimensionality. To further understand the final selection of important features, all features were compared across the three target labels and summarized using an ANOVA for numerical data and a Chi-squared test for categorical data. The same procedure was applied across the different participant cohorts (i.e., HC, CRPS, LBP, and SCI). By comparing feature distributions across cohorts, we aimed to identify systematic differences that may influence the model’s performance through cohort-specific characteristics rather than by pain intensity-related features.

The best base classification model was identified by ranking various algorithms based on the F1-score, which represents the harmonic mean of precision and recall^38^. The interpretation of F1 scores varies by context and lacks universally defined thresholds. However, general guidelines suggest ≥ 0.90 excellent performance, 0.80–0.89 good performance, 0.70–0.79 average performance, and < 0.70 poor performance. The base models were ranked based on the mean F1-score of the 10-folds minus one standard deviation. Using this approach, we were able to choose the best performing base model with the lowest variability. In cases where different models performed best for the QST-, biopsychosocial-, and noQST model, we applied all top-performing models to all data sets. This approach enabled a more thorough comparison of model performance.

The hyperparameters of the top-ranked model were optimized using random grid search. To evaluate the final model’s performance, we collected the predicted and ground truth labels from the test set of each cross-validated tenfold run. We assessed performance based on overall accuracy, precision, recall, and F1-score. Additionally, we computed a confusion matrix and plotted the Receiver Operating Characteristic (ROC) curve and Precision-Recall (PR) curve for each target label separately. Finally, we extracted the feature importance coefficients for each target label and estimated the overall contribution of each feature to the model’s performance by summing the absolute importance weight of each label and averaging across all ten folds.

## Results

### Study population

A total of 101 participants with chronic pain and 63 age- and sex-matched HC were recruited for this study. The patient group comprised of 21 participants with CRPS, 61 participants with LBP, and 19 participants with neuropathic pain after SCI. Out of the 164 participants initially enrolled, seven had to be excluded from the study. One participant with CRPS was unable to tolerate the study protocol, and three patients (one with LBP, two with SCI) and two HC were excluded after sensory abnormalities were identified during sensory bedside testing. One participant with LBP was excluded due to a suspected, previously unrecognized psychiatric condition. Differences between the participant cohorts regarding all collected features can be found in the supplementary Table S.1.

Regarding pain intensity, the study population included 61 participants with “no pain” (i.e., 61 HC), 42 participants with “mild-to-moderate pain” (i.e., 5 CRPS, 32 LBP, and 5 SCI), and 53 participants with “severe pain” (i.e., 15 CRPS, 27 LBP, 11 SCI). Differences in features across the groups depending on the target label can be found in the supplementary Table S.2.

### Classification performance comparison

**Fig. 2:**
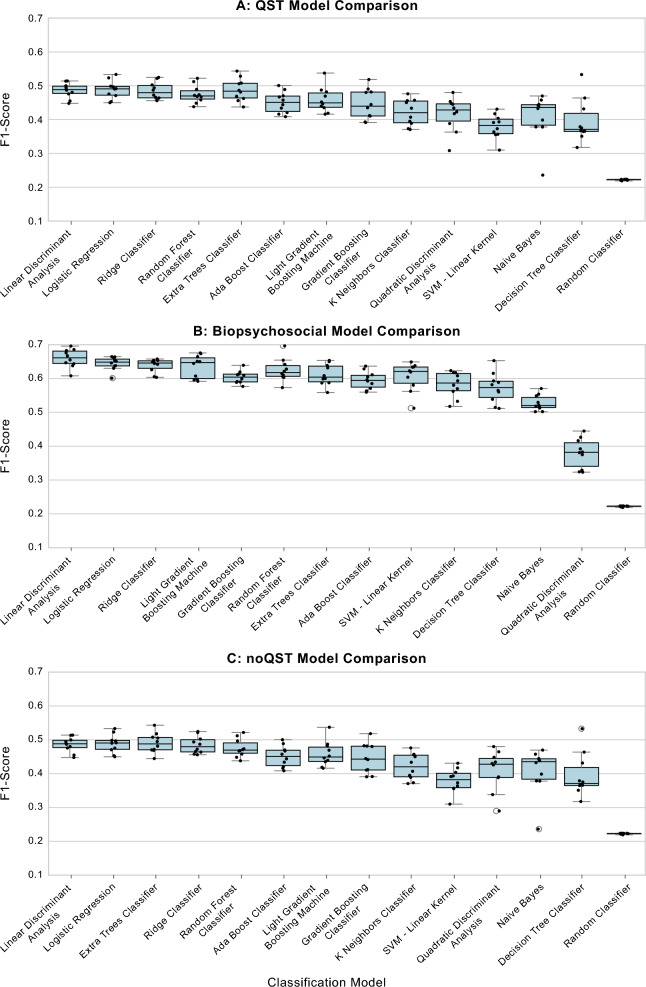
Base model comparison of the (**A**) QST model and (**B**) Biopsychosocial model (**C**) noQST model. The F1-score of the 10-folds are illustrated for each classification model integrated in the pycaret package. The models are ordered based on the mean—standard deviation from left to right to evaluate the best performing base model.

Figure 2 illustrates the base model performance ranked by the mean F1-score minus one standard deviation for both the QST-, biopsychosocial-, and noQST model separately. Based on these results, hyperparameter tuning was conducted on the linear discriminant analysis (LDA), which exhibited the highest score for all data sets.

**Fig. 3:**
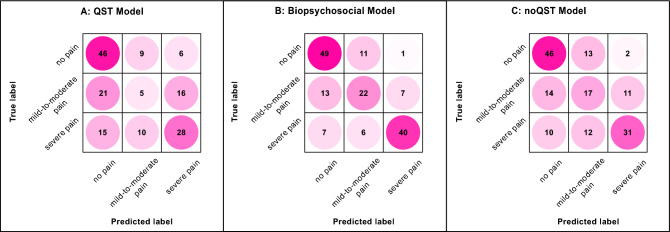
Confusion matrix of the classification QST- (left), biopsychosocial model (middle), and noQST model (right). Each cell shows the number of participants, also indicated by the shading intensity: more intense pink shades represent a higher number of participants in that specific cell.

**Fig. 4:**
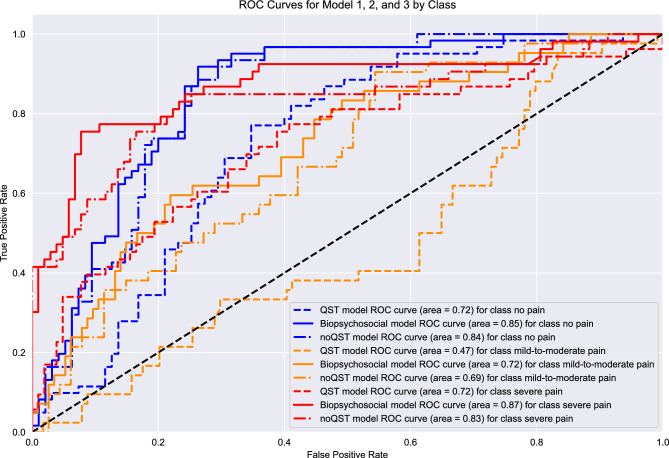
Receiver Operating Characteristic (ROC) curves for the QST- (dashed curve), biopsychosocial- (solid curve), and noQST model (dash-dotted curve) for each pain intensity class. The “no pain” class is represented in blue, the “mild-to-moderate pain” class in yellow, and the “severe pain” class in red. AUC: area under curve.

**Fig. 5:**
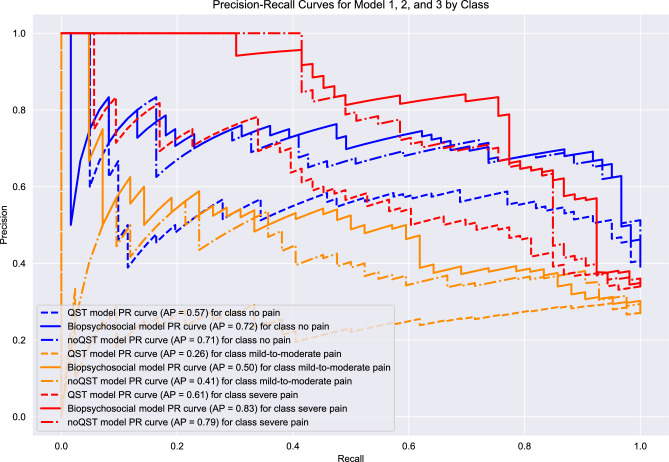
Precision-Recall curve of the QST- (dashed curve), biopsychosocial- (solid curve), and noQST model (dash-dotted curve) for each pain intensity class. The “no pain” class is represented in blue, the “mild-to-moderate pain” class in yellow, and the “severe pain” class in red. AP: average precision.

The final model performances of the linear discriminant analysis from the tenfold cross-validation are presented in Table 1, including performance metrics for each target label separately. Figure 3 shows the confusion matrix of all models. Figure 4 illustrates the ROC curves for the three pain intensity classes for all classification models, along with the area under the curve (AUC). The Precision-Recall curve for each model and class is shown in Fig. 5, including the average precision for each curve.

**Table 1:** Linear discriminant analysis metrics of the QST-, biopsychosocial-, and noQST model.

QST Model				
Label	Accuracy	Recall	Precision	F1-Score
No Pain	0.67	0.75	0.56	0.64
Mild-to-Moderate Pain	0.64	0.12	0.21	0.15
Severe Pain	0.70	0.53	0.56	0.54
Overall	0.51	0.51	0.47	0.48

Biopsychosocial Model				
Label	Accuracy	Recall	Precision	F1-Score
No Pain	0.80	0.80	0.71	0.75
Mild-to-Moderate Pain	0.76	0.52	0.56	0.54
Severe Pain	0.87	0.75	0.83	0.79
Overall	0.71	0.71	0.71	0.71

noQST Model				
Label	Accuracy	Recall	Precision	F1-Score
No Pain	0.75	0.75	0.66	0.70
Mild-to-Moderate Pain	0.68	0.40	0.40	0.40
Severe Pain	0.78	0.58	0.70	0.64
Overall	0.60	0.61	0.60	0.60

Table 2 provides a comprehensive summary of feature importance for the QST model training process, ranked from the most important feature (on the top) to the least important feature (at the bottom). A feature is included in Table 2 if it ranks among the top 70% most important features in at least one of the ten folds. The weight represents the average absolute importance across the 10-folds ± the standard deviation, offering a robust measure of each feature’s overall contribution to the model. The ‘number of occurrences’ indicates how many of the 10-folds identified the feature as one of the nine most important. Tables 3 and 4 present the same results for the biopsychosocial-and noQST model respectively. Similarly to Table 2, A feature is included in Table 3 or if it ranks among the top 20% (biopsychosocial) or 30% (noQST) most important features in at least one of the ten folds. The direction of feature importance of Tables 2, 3 and 4 is detailed in Supplementary Tables S1 and S2.

**Table 2:** Feature importance of the classification model trained on sensory profile features of the most painful area (QST).

Feature	Weight	# of occurrences in 10-folds
WDT	24.9 ± 13.5	9
MPT	22.9 ± 13.3	8
PPT	22.4 ± 8.7	10
CDT	22.2 ± 10.7	10
VDT	21.3 ± 9.5	10
MDT	15.7 ± 9.9	10
MPS	14.6 ± 12.7	9
CPT	4.7 ± 9.1	4
WUR	4.3 ± 8.7	3
HPT	4.2 ± 8.6	3
PHS	3.8 ± 1.9	9
TSL	2.6 ± 8.3	1
DMA	1.1 ± 1.7	4

The weight indicates the average absolute feature importance ± standard deviation across the 10-folds. The features are ranked from most important feature (top) to least important feature (bottom). The # occurrences in 10-folds indicates how many of the 10-folds selected the given feature as one of the nine most important features.

CDT: cold detection threshold, CPT: cold pain threshold, DMA: dynamic mechanical allodynia, HPT: heat pain threshold, MDT: mechanical detection threshold, MPS: mechanical pain sensitivity, MPT: mechanical pain threshold, PHS: paradoxical heat sensation, PPT: pressure pain threshold, TSL: thermal sensory limen, VDT: vibration detection threshold, WDT: warm detection threshold, WUR: wind-up ratio.

**Table 3:** Feature importance of the classification model trained on all biopsychosocial features.

Feature	Weight	# of occurrences in 10-folds
Quality of life (very good)	14.8 ± 27.6	10
VDT most painful area	14.6 ± 8.6	10
HADS (depression)	11.8 ± 13.6	10
PCS	10.9 ± 8.9	10
Quality of life (moderate)	9.0 ± 19.3	9
Quality of life (good)	8.9 ± 28.1	1
HADS (anxiety)	8.3 ± 6.7	7
CDT most painful area	7.9 ± 15.6	3
Fatigue (no problems)	6.9 ± 3.2	10
Fatigue (moderate problems)	5.6 ± 4.9	9
Fatigue (severe problems)	5.5 ± 4.9	9
General health (good)	1.4 ± 3.0	2

The weight indicates the average absolute feature importance ± standard deviation across the 10-folds. The # occurrences in 10-folds indicates how many of the 10-folds selected the given feature as one of the nine most important features.

CDT: cold detection threshold, HADS: hospital anxiety and depression scale, PCS: pain catastrophizing scale, VDT: vibration detection threshold.

**Table 4:** Feature importance of the classification model trained on all noQST features.

Feature	Weight	# of occurrences in 10-folds
Quality of life (very good)	15.7 ± 24.7	10
HADS (anxiety)	14.3 ± 5.4	10
HADS (depression)	12.9 ± 13.7	10
PCS	11.7 ± 7.0	10
Quality of life (moderate)	9.5 ± 17.5	10
Quality of life (good)	8.1 ± 24.2	1
Fatigue (no problems)	7.4 ± 2.7	10
Fatigue (moderate problems)	6.0 ± 3.7	10
Fatigue (severe problems)	4.4 ± 3.9	10
General health (good)	1.8 ± 2.5	4
General health (less good)	0.8 ± 1.7	2
Quality of life (bad)	0.5 ± 0.8	3

The weight indicates the average absolute feature importance ± standard deviation across the 10-folds. The # occurrences in 10-folds indicates how many of the 10-folds selected the given feature as one of the nine most important features.

HADS: hospital anxiety and depression scale, PCS: pain catastrophizing scale.

## Discussion

The present study aimed to use advanced classification methods to better understand differences in chronic pain intensity across various conditions by incorporating biological, psychological, and social features compared to focusing solely on sensory profiles. This approach enables the identification of the most influential and common factors associated with perceived chronic pain intensity regardless of the clinical diagnosis.

We found that all models (i.e., the QST-, biopsychosocial-, and noQST model) performed better than a random classifier (Table 1), suggesting that all models contain valuable features in distinguishing the three pain classes (i.e., no pain, mild-to-moderate pain, and severe pain). The noQST model outperformed the QST model (Table 1); however, its performance did not reach that of the full biopsychosocial model that includes all features (i.e., including QST). This finding is consistent with our observation that several QST features remain among the most important predictors in the biopsychosocial model (Table 3). Together, these results indicate that while non-sensory biopsychosocial factors account for a substantial proportion of the classification performance, the sensory profile still provides valuable additional information, likely reflecting underlying mechanisms associated with more severe chronic pain^1^. Overall, these findings align with the widely accepted view that a complex interplay between biological, psychological and social factors influences a patient’s spontaneous chronic pain^12^.

The model performance evaluated separately for each target label (Table 1) revealed that both the “no pain” and “severe pain” classes were generally easier to identify, regardless of the feature set used. However, for the “mild-to-moderate pain” class, the biopsychosocial model notably improved the F1-score from 0.15 (QST model) or 0.40 (noQST model) to 0.54. This suggests that the sensory profile of the most painful area may be sufficient to distinguish participants with no pain from those with severe pain but may fail to capture mild-to-moderate pain. Here, the biopsychosocial model outperformed the QST model, highlighting that factors beyond somatosensory function are needed to discriminate more nuanced gradations of chronic pain severity (i.e., mild-to-moderate). This aligns with previous findings, where the sensory profile of the most painful area alone did not effectively differentiate participants with differing spontaneous pain intensities^9,39,40^.

The findings of our study underscore the potential significance of sensory loss of function in the development and persistence of severe pain (Table 2). Participants experiencing more severe pain demonstrated greater loss of function (Table S2). Loss of function may be less frequently observed than gain of function in participants without pain (i.e., HC), as suggested by our previous work^9^. This finding might additionally be driven by participants with neuropathic pain after SCI, where loss of function is the defining feature of the spinal lesion. Nevertheless, loss of function in the most painful areas could be a key contributor to severe pain, possibly resulting from pain-induced hypoalgesia^41–43^ or deafferentation^44–48^. While the mechanism of deafferentation pain is not entirely understood, it likely arises from hyperactive neurons in the spinal cord^44^ and thalamus^47,48^, and/or from cortical reorganization of the somatosensory and motor cortex due to reduced afferent input^45,46^.

In addition to psychophysical assessments, we included neurophysiological measures (i.e., CHEP, SSR and SSEP), which provide more objective insights of the functional integrity of the spinothalamic/-reticular tract (CHEP and SSR) and dorsal columns (SSEP). However, these measures did not emerge among the most important features in the classification models. This may partly reflect the heterogeneous nature of the cohort, comprising both individuals with and without structural spinal tract lesions. In patients with SCI, lesion severity is often not directly linked to the occurrence or severity of chronic pain^49^. As CHEPs, SSR, and SSEPs primarily reflect abnormalities in ascending sensory pathways rather than mechanisms driving chronic pain, these neurophysiological measures may show limited sensitivity to interindividual differences in perceived chronic pain intensity. A more promising marker of underlying pathomechanisms associated with chronic pain intensity is experimental pain habituation, (i.e., reduction in neurophysiological responses during repeated stimulation). However, although alterations in experimental pain habituation have been associated with chronic pain intensity within specific chronic pain etiologies, including SCI^21^, such associations have not been consistently observed across diverse pain conditions^27^. Consequently, the absence of these neurophysiological measures among the most informative predictors does not negate their physiological relevance. Rather, it suggests that objective measures of sensory processing may be less sensitive for predicting chronic pain severity than psychophysical assessments of sensory function (e.g., sensory profiles or quantitative sensory testing).

Sensory gain of function, particularly for mechanical stimuli, was also represented by multiple features within the set of most important features. Mechanical hyperalgesia in the most painful area in the absence of thermal hyperalgesia, suggests that spinal sensitization may play a significant role in the development and maintenance of chronic pain^43,50^. This finding might be driven by participants with CRPS, as 75% of participants with CRPS reported severe pain and typically exhibit pronounced mechanical hyperalgesia, i.e., reduced PPTs^51–54^. Although the comparable distribution of severe pain across the patient cohorts (CRPS N = 15, LBP N = 27, SCI N = 11) helps mitigate concerns about cohort-specific bias (Table S1), the uneven distribution of pain severity within individual cohorts may still introduce bias into the observed results.

An interesting observation was that the biopsychosocial model also included some sensory profile features (e.g., VDT, CDT) among its most important features. To explore whether this finding might be biased by the individuals with SCI we assessed the frequency of loss of function in VDT (as this was one of the most important features, Table 3) within the individuals with CRPS and LBP separately. In the mild-to-moderate pain group none of the five CRPS patients (0%) and only three of the 32 LBP patients (9%) showed a relevant loss of function (defined as a z-score < -1.96). Within the severe pain group seven out of 15 CRPS patients (47%) and eight out of 27 LBP patients (30%) showed a significant loss of function in VDT, indicating that this feature might be a relevant contributor of severe pain regardless of the clinical diagnosis. Nevertheless, the set of selected features leans more heavily towards the extended list of biopsychosocial features (e.g., HADS, PCS, quality of life, fatigue). Similar features have also been shown to predict the spatial spread of chronic pain in previous studies^14^. A key challenge in interpreting these features, however, is the inability to infer causality. For instance, sleep quality, which might be related to the presence of fatigue, has been shown to be affected bidirectionally: sleep disturbances can predict chronic pain, while chronic pain can also lead to sleep disturbances^55^. Still, it has been previously noted^56^ that sleep disturbances are a more significant predictor of chronic pain than the reverse relationship. This bidirectional influence similarly applies to psychosocial factors, such as a depressed mood, with systematic reviews of chronic pain conditions underscoring the link between psychosocial factors and heightened pain intensity^57–60^. For instance, in individuals with CRPS, a prospective study^61^ revealed that increased pain led to heightened levels of depression, anxiety, and anger the following day, while depressed mood also contributed to greater pain the next day. Although the reciprocal relationships between psychosocial factors and chronic pain remain incompletely understood, they highlight the need of therapeutic interventions that embrace the biopsychosocial model of pain. Such approaches are best implemented through interdisciplinary pain management programs, where healthcare professionals from various disciplines (such as physicians, psychologists, physical therapists, and social workers) work together within the same institution^62^. This integrated care model necessitates active communication between departments, aiming at holistic and coordinated treatment strategies that address the multifaceted nature of chronic pain.

Although various complementary experimental pain paradigms were included to capture peripheral, spinal, and supra-spinal sensitization, as well as pathological top-down modulation processes in chronic pain, none emerged as the most important features in the analysis. Interestingly, most of the key features were related to questionnaires, challenging the relevance of identifying chronic pain mechanisms using complementary experimental paradigms, which are typically time-consuming and difficult to measure in a large study sample. Additionally, findings from complementary experimental pain paradigms may not be consistent across chronic pain conditions and thus, not emerge as universally important feature in our heterogenous study sample. This aligns with previous findings from our group (using the same study sample), where, for example, experimental pain habituation was not found to be reduced in individuals with different chronic pain conditions compared to HC^27^. Nonetheless, these experimental pain paradigms can still provide valuable insights regarding potential underlying mechanisms, particularly when evaluated within a specific patient cohort or used to identify subtle variations in chronic pain intensity.

One limitation of this study is the relatively small sample size, which may affect the generalizability of our findings. Moreover, our study sample lacked participants with medical conditions without chronic pain (e.g., SCI without neuropathic pain), whose inclusion could have provided valuable comparative insights into the underlying mechanisms of chronic pain. However, since the primary goal was to identify the most common features relevant for spontaneous pain intensity across a heterogeneous sample of chronic pain conditions the absence of pain-free medical conditions is unlikely to have significantly influenced the overall findings. Furthermore, the categorization of certain factors into biological, psychological, or social domains may not always be distinct, as some variables can span multiple domains. Finally, much of the data relies on self-reported questionnaires, which may introduce recall bias. In addition, some factors may not have emerged as among the most important variables for classifying the chronic pain group because they were assessed using brief or single-item measures (e.g., general health and fatigue).

To conclude, a key strength of this study is the inclusion of advanced classification methods, multiple chronic pain cohorts, and experimental pain paradigms alongside psychological and social factors, allowing for a comprehensive analysis of factors influencing chronic pain intensity across diverse populations. By incorporating psychological and social dimensions, we enhanced the ability to differentiate between participants with “no pain”, “mild-to-moderate pain”, and “severe pain”. Key contributing factors potentially explaining the variance in spontaneous pain intensity included quality of life, loss of sensation, depressive mood, pain catastrophizing, anxiety, fatigue, and general health. These findings highlight the necessity of an interdisciplinary treatment approach that integrates the expertise of, for example, physicians, psychologists, physical therapists, and social workers to effectively address the complex and multifactorial nature of chronic pain.

## Supplementary Information

Supplementary Information.

## Data Availability

Data and programming code are available from the corresponding author upon reasonable request.
