# Supplementary material for: The role of biopsychosocial factors in classifying pain intensity across various chronic pain conditions
Source: Sci Rep. 2026 May 29;16:24867. doi: 10.1038/s41598-026-46612-9 (PMC13457856; doi:10.1038/s41598-026-46612-9)
Supplement: Supplementary file 1 [file 41598_2026_46612_MOESM1_ESM.docx]

Table S1: Feature evaluation separated by participant cohort.

| **Feature** | **% missing** | **Test** | **Cohort HC** | **Cohort CRPS** | **Cohort LBP** | **Cohort SCI** | **p-value** | | **Adjusted**  **p-value (Bonferroni)** | **Statistic** |
| --- | --- | --- | --- | --- | --- | --- | --- | --- | --- | --- |
| Age [y] | 0.00 | ANOVA | 48.38 (16.05) | 45.05 (13) | 50.81 (16.64) | 56.63 (9.43) | 0.126 | 1.000 | | 1.94 |
| CHEP: habituation control area [% change] | 9.62 | ANOVA | -19.02 (28.14) | -18.08 (40.46) | -21.25 (28.13) | -19.21 (34.48) | 0.975 | 1.000 | | 0.07 |
| CHEP: habituation most painful area [% change] | 23.72 | nt | nt | nt | nt | nt | nt | nt | | nt |
| CHEP: integrity control area [pathological/physiological] | 6.41 | Chi-Squared | 5/54 (8/92) | 4/13 (24/76) | 8/47 (15/85) | 4/11 (27/73) | 0.880 | 1.000 | | 0.48 |
| CHEP: integrity most painful area [pathological/physiological] | 11.54 | nt | nt | nt | nt | nt | nt | nt | | nt |
| CPM control area [% change] | 5.77 | ANOVA | -27.54 (30) | -26.51 (39.6) | -21.46 (29.74) | -39.23 (38.37) | 0.291 | 1.000 | | 1.26 |
| CPM most painful area [% change] | 16.03 | nt | nt | nt | nt | nt | nt | nt | | nt |
| Education [compulsory schooling/apprenticeship/secondary school/higher technical college/university of applied sciences/university ] | 0.00 | Chi-Squared | 0/13/5/9/11/23 (0/21/8/15/18/38) | 0/14/0/3/0/3 (0/70/0/15/0/15) | 2/24/5/7/5/16 (3/41/8/12/8/27) | 0/9/2/2/2/1 (0/56/13/13/13/6) | 0.000 | 0.017* | | 18.09 |
| General health [excellent/very good/good/less good/bad] | 0.00 | Chi-Squared | 15/36/10/0/0 (25/59/16/0/0) | 0/4/9/6/1 (0/20/45/30/5) | 4/23/28/4/0 (7/39/47/7/0) | 2/4/10/0/0 (13/25/63/0/0) | 0.221 | 1.000 | | 4.40 |
| HADS (anxiety) [score] | 0.00 | ANOVA | 3.43 (2.51) | 8.05 (3.86) | 5.03 (3.4) | 4.19 (3.37) | 0.000 | 0.000* | | 11.26 |
| HADS (depression) [score] | 0.00 | ANOVA | 1.69 (1.94) | 6.8 (5.02) | 3.85 (3.3) | 4.5 (3.79) | 0.000 | 0.000* | | 14.39 |
| IPAQ [low/moderate/high] | 0.00 | Chi-Squared | 5/31/25 (8/51/41) | 4/6/10 (20/30/50) | 9/22/28 (15/37/47) | 7/4/5 (44/25/31) | 0.580 | 1.000 | | 1.97 |
| Life quality [very good/good/moderate/bad/very bad ] | 0.00 | Chi-Squared | 34/24/3/0/0 (56/39/5/0/0) | 0/3/12/4/1 (0/15/60/20/5) | 12/31/12/4/0 (20/53/20/7/0) | 2/7/5/2/0 (13/44/31/13/0) | 0.001 | 0.050 | | 15.87 |
| MAIA [percentile] | 0.00 | ANOVA | 56.67 (19.52) | 43.81 (17.15) | 52.46 (18.23) | 55.72 (17.65) | 0.059 | 1.000 | | 2.54 |
| Pain label [no pain/mild-to-moderate pain/severe pain] | 0.00 | Chi-Squared | 61/0/0 (100/0/0) | 0/5/15 (0/25/75) | 0/32/27 (0/54/46) | 0/5/11 (0/31/69) | 0.000 | 0.000* | | 96.72 |
| PCS [score] | 0.00 | ANOVA | 5.43 (6.85) | 22.85 (11.98) | 12.9 (9.74) | 13.13 (8.33) | 0.000 | 0.000* | | 20.86 |
| Perceived pain habituation control area [% change] | 5.13 | ANOVA | -10.88 (20.67) | -12.97 (24.82) | -17.63 (32.71) | -23.81 (18.85) | 0.275 | 1.000 | | 1.30 |
| Perceived pain habituation most painful area [% change] | 8.33 | ANOVA | -18.28 (20.54) | -5.23 (20.8) | -21.67 (19.38) | -18.39 (39.63) | 0.061 | 1.000 | | 2.52 |
| QST: Cold detection threshold most painful area [z-score] | 1.28 | ANOVA | -0.23 (0.81) | -1 (1.29) | -0.67 (0.87) | -2.76 (1.43) | 0.000 | 0.000* | | 26.18 |
| QST: Cold pain threshold control area [z-score] | 2.56 | ANOVA | 0.05 (1.09) | 0.35 (1.02) | -0.16 (1.11) | 0.26 (1.51) | 0.322 | 1.000 | | 1.17 |
| QST: Cold pain threshold most painful area [z-score] | 0.00 | ANOVA | 0.22 (1.05) | 0.63 (1.02) | -0.02 (1.01) | -0.79 (0.56) | 0.000 | 0.011* | | 6.75 |
| QST: Dynamic mechanical allodynia control area [z-score] | 2.56 | ANOVA | 0.08 (0.62) | 0 (0) | 0.03 (0.19) | 0.02 (0.07) | 0.845 | 1.000 | | 0.27 |
| QST: Dynamic mechanical allodynia most painful area [z-score] | 0.00 | ANOVA | 0.08 (0.56) | 0.55 (2.24) | 0.15 (0.61) | 0.04 (0.12) | 0.256 | 1.000 | | 1.36 |
| QST: Heat pain threshold control area [z-score] | 2.56 | ANOVA | 0.1 (1.17) | 0.07 (0.95) | -0.07 (1.14) | 0.26 (1.17) | 0.713 | 1.000 | | 0.46 |
| QST: Heat pain threshold most painful area [z-score] | 0.00 | ANOVA | 0.45 (1.16) | 0.79 (1.35) | 0.45 (1.12) | -1.17 (0.8) | 0.000 | 0.000* | | 10.75 |
| QST: Mechanical detection threshold most painful area [z-score] | 1.28 | ANOVA | -1.07 (0.87) | -1.54 (1.36) | -1.37 (1.03) | -3.29 (0.98) | 0.000 | 0.000* | | 18.27 |
| QST: Mechanical pain sensitivity control area [z-score] | 2.56 | ANOVA | 1.14 (1.21) | 1.43 (1.81) | 1.42 (1.27) | 0.71 (1.16) | 0.223 | 1.000 | | 1.48 |
| QST: Mechanical pain sensitivity most painful area [z-score] | 0.00 | ANOVA | 1.27 (1.03) | 2.13 (1.26) | 1.62 (1.24) | -0.89 (1.4) | 0.000 | 0.000* | | 23.06 |
| QST: Mechanical pain threshold control area [z-score] | 2.56 | ANOVA | 1.3 (1.24) | 0.8 (1.3) | 1.13 (1.24) | 1.06 (1.46) | 0.540 | 1.000 | | 0.72 |
| QST: Mechanical pain threshold most painful area [z-score] | 0.00 | ANOVA | 1.06 (1.14) | 1.71 (1.13) | 0.7 (0.98) | -1.47 (1.98) | 0.000 | 0.000* | | 24.22 |
| QST: Paradoxical heat sensation most painful area [z-score] | 1.28 | ANOVA | 0.15 (0.48) | 0.25 (0.79) | 0.03 (0.26) | 0.14 (0.53) | 0.293 | 1.000 | | 1.25 |
| QST: Pressure pain threshold control area [z-score] | 2.56 | ANOVA | 0.48 (1.26) | 1.58 (1.41) | 0.58 (1.55) | 0.95 (1.5) | 0.034 | 1.000 | | 2.97 |
| QST: Pressure pain threshold most painful area [z-score] | 0.00 | ANOVA | -0.12 (1.46) | 3.08 (1.56) | 0.2 (1.4) | -0.77 (1.33) | 0.000 | 0.000* | | 29.59 |
| QST: Thermal sensory limen most painful area [z-score] | 1.28 | ANOVA | -0.46 (0.84) | -1.15 (1.01) | -0.47 (1.06) | -2.29 (1.22) | 0.000 | 0.000* | | 15.64 |
| QST: Vibration detection threshold most painful area [z-score] | 1.28 | ANOVA | -0.28 (1.19) | -1.47 (1.44) | -0.83 (1.69) | -3.22 (1.37) | 0.000 | 0.000* | | 16.69 |
| QST: Warm detection threshold most painful area [z-score] | 1.28 | ANOVA | -0.03 (1.07) | -0.58 (1.25) | -0.09 (1.24) | -1.86 (1.26) | 0.000 | 0.000* | | 10.28 |
| QST: Wind-up ratio control area [z-score] | 2.56 | ANOVA | 0.07 (0.99) | 0.36 (1.5) | -0.04 (1) | 0.01 (1.36) | 0.625 | 1.000 | | 0.59 |
| QST: Wind-up ratio most painful area [z-score] | 0.00 | ANOVA | 0.13 (1.08) | 0.92 (1.66) | 0.05 (1.17) | -2.08 (1.79) | 0.000 | 0.000* | | 17.79 |
| Relationship status [single/in a committed relationship-married/separated-divorced/widowed] | 0.00 | Chi-Squared | 22/36/3/0 (36/59/5/0) | 8/10/2/0 (40/50/10/0) | 20/31/7/1 (34/53/12/2) | 3/9/4/0 (19/56/25/0) | 0.779 | 1.000 | | 1.09 |
| Sex [female/male] | 0.00 | Chi-Squared | 34/27 (56/44) | 17/3 (85/15) | 37/22 (63/37) | 2/14 (13/88) | 0.036 | 1.000 | | 8.55 |
| SSEP [pathological/physiological] | 8.97 | Chi-Squared | 4/53 (7/93) | 1/15 (6/94) | 7/50 (12/88) | 8/4 (67/33) | 0.000 | 0.000* | | 26.41 |
| SSR habituation control area [% change] | 14.10 | nt | nt | nt | nt | nt | 0.702 | 1.000 | | nt |
| SSR habituation most painful area [% change] | 10.90 | nt | nt | nt | nt | nt | 0.484 | 1.000 | | nt |
| Tiredness [no problems/slight problems/moderate problems/severe problems] | 0.00 | Chi-Squared | 34/26/1/0 (56/43/2/0) | 0/4/10/6 (0/20/50/30) | 12/25/18/4 (20/42/31/7) | 3/7/4/2 (19/44/25/13) | 0.000 | 0.000* | | 44.26 |
| TSP control area [% change] | 1.28 | ANOVA | 14.51 (24.67) | 15.02 (39.94) | 9.47 (20.21) | 7.35 (29.42) | 0.599 | 1.000 | | 0.63 |
| TSP most painful area [% change] | 7.05 | ANOVA | 8.42 (20.24) | 21.86 (19.53) | 11.07 (16.98) | 14.83 (39.99) | 0.089 | 1.000 | | 2.21 |

*Numeric data is shown as mean (standard deviation) and categorical data is shown as count (% of group). Abbreviations: CHEP: contact-heat evoked potential, CPM: conditioned pain modulation, CRPS: complex regional pain syndrome, HADS: hospital anxiety and depression scale, HC: healthy control, IPAQ-SF: international physical activity questionnaire – short form, LBP: low back pain, MAIA: multidimensional assessment of interoceptive awareness, nt: not tested (due to more than 10% missing data), PCS: pain catastrophizing scale, QST: quantitative sensory testing, SCI: spinal cord injury, SSEP: somatosensory evoked potential, SSR: sympathetic skin response, TSP: temporal summation of pain, *significant after adjustment*

Table S2: Feature evaluation separated by target label.

| **Feature** | **% missing** | **Test** | **Label *no pain* (N=61)** | **Label *mild-to-moderate* (N=42)** | **Label *severe pain* (N=53)** | **p-value** | **Adjusted**  **p-value (Bonferroni)** | **Statistic** |
| --- | --- | --- | --- | --- | --- | --- | --- | --- |
| Age [y] | 0.00 | ANOVA | 48.38 (16.05) | 52.98 (15.23) | 48.68 (15.11) | 0.283 | 1.000 | 1.27 |
| CHEP habituation control area (block 1) [% change] | 9.62 | ANOVA | -19.02 (28.14) | -22.24 (28.53) | -18.68 (34.19) | 0.844 | 1.000 | 0.17 |
| CHEP habituation most painful area (block 1) [% change] | 23.72 | nt | nt | nt | nt | nt | nt | nt |
| CHEP: integrity control area [pathological/physiological] | 6.41 | Chi-Squared | 5/54 (8/92) | 6/33 (15/85) | 10/38 (21/79) | 0.787 | 1.000 | 0.48 |
| CHEP: integrity most painful area [pathological/physiological] | 11.54 | nt | nt | nt | nt | nt | nt | nt |
| Cohort [HC/CRPS/LBP/SCI] | 0.00 | Chi-Squared | 61/0/0/0 (100/0/0/0) | 0/5/32/5 (0/12/76/12) | 0/15/27/11 (0/28/51/21) | 0.001 | 0.021* | 15.18 |
| CPM control area [% change] | 5.77 | ANOVA | -27.54 (30) | -27.7 (33.35) | -23.86 (34.2) | 0.802 | 1.000 | 0.22 |
| CPM most painful area [% change] | 16.03 | nt | nt | nt | nt | nt | nt | nt |
| Education [compulsory schooling/apprenticeship/secondary school/higher technical college/university of applied sciences/university ] | 0.00 | Chi-Squared | 0/13/9/5/11/23 (0/21/15/8/18/38) | 0/18/4/5/3/12 (0/43/10/12/7/29) | 2/29/8/2/4/8 (4/55/15/4/8/15) | 0.001 | 0.050 | 13.40 |
| General health [excellent/very good/good/less good/bad] | 0.00 | Chi-Squared | 15/36/10/0/0 (25/59/16/0/0) | 3/18/20/1/0 (7/43/48/2/0) | 3/13/27/9/1 (6/25/51/17/2) | 0.932 | 1.000 | 0.14 |
| HADS (anxiety) [score] | 0.00 | ANOVA | 3.43 (2.51) | 4.4 (3.22) | 6.42 (3.87) | 0.000 | 0.000* | 12.52 |
| HADS (depression) [score] | 0.00 | ANOVA | 1.69 (1.94) | 2.55 (2.19) | 6.19 (4.27) | 0.000 | 0.000* | 34.64 |
| IPAQ-SF[low/moderate/high] | 0.00 | Chi-Squared | 5/31/25 (8/51/41) | 9/10/23 (21/24/55) | 11/22/20 (21/42/38) | 0.096 | 1.000 | 4.68 |
| Life quality [very good/good/moderate/bad/very bad ] | 0.00 | Chi-Squared | 34/24/3/0/0 (56/39/5/0/0) | 7/27/7/1/0 (17/64/17/2/0) | 7/14/22/9/1 (13/26/42/17/2) | 0.000 | 0.002* | 19.87 |
| MAIA [percentile] | 0.00 | ANOVA | 56.67 (19.52) | 51.69 (19.51) | 50.79 (17.22) | 0.203 | 1.000 | 1.61 |
| PCS [score] | 0.00 | ANOVA | 5.43 (6.85) | 10.79 (8.99) | 18.4 (10.87) | 0.000 | 0.000* | 29.81 |
| Perceived pain habituation control area [% change] | 5.13 | ANOVA | -10.88 (20.67) | -13 (33.08) | -21.64 (25.27) | 0.088 | 1.000 | 2.47 |
| Perceived pain habituation most painful area [% change] | 8.33 | ANOVA | -18.28 (20.54) | -15.74 (23.56) | -19.28 (24.13) | 0.767 | 1.000 | 0.27 |
| QST: Cold detection threshold most painful area [z-score] | 1.28 | ANOVA | -0.23 (0.81) | -0.98 (1.02) | -1.11 (1.46) | 0.000 | 0.004* | 9.95 |
| QST: Cold pain threshold control area [z-score] | 2.56 | ANOVA | 0.05 (1.09) | -0.02 (1.15) | 0.03 (1.22) | 0.954 | 1.000 | 0.05 |
| QST: Cold pain threshold most painful area [z-score] | 0.00 | ANOVA | 0.22 (1.05) | -0.02 (1.02) | -0.01 (1.07) | 0.388 | 1.000 | 0.95 |
| QST: Dynamic mechanical allodynia control area [z-score] | 2.56 | ANOVA | 0.08 (0.62) | 0.01 (0.05) | 0.03 (0.21) | 0.678 | 1.000 | 0.39 |
| QST: Dynamic mechanical allodynia most painful area [z-score] | 0.00 | ANOVA | 0.08 (0.56) | 0.11 (0.4) | 0.3 (1.47) | 0.424 | 1.000 | 0.86 |
| QST: Heat pain threshold control area [z-score] | 2.56 | ANOVA | 0.1 (1.17) | -0.06 (1.16) | 0.07 (1.08) | 0.754 | 1.000 | 0.28 |
| QST: Heat pain threshold most painful area [z-score] | 0.00 | ANOVA | 0.45 (1.16) | 0.14 (1.21) | 0.33 (1.36) | 0.454 | 1.000 | 0.79 |
| QST: Mechanical detection threshold most painful area [z-score] | 1.28 | ANOVA | -1.07 (0.87) | -1.56 (1.19) | -1.81 (1.35) | 0.003 | 0.119 | 6.07 |
| QST: Mechanical pain sensitivity control area [z-score] | 2.56 | ANOVA | 1.14 (1.21) | 1.01 (1.17) | 1.53 (1.5) | 0.126 | 1.000 | 2.10 |
| QST: Mechanical pain sensitivity most painful area [z-score] | 0.00 | ANOVA | 1.27 (1.03) | 1 (1.44) | 1.55 (1.72) | 0.175 | 1.000 | 1.76 |
| QST: Mechanical pain threshold control area [z-score] | 2.56 | ANOVA | 1.3 (1.24) | 0.93 (1.25) | 1.17 (1.32) | 0.363 | 1.000 | 1.02 |
| QST: Mechanical pain threshold most painful area [z-score] | 0.00 | ANOVA | 1.06 (1.14) | 0.33 (1.46) | 0.72 (1.65) | 0.040 | 1.000 | 3.28 |
| QST: Paradoxical heat sensation most painful area [z-score] | 1.28 | ANOVA | 0.15 (0.48) | 0.17 (0.63) | 0.04 (0.28) | 0.329 | 1.000 | 1.12 |
| QST: Pressure pain threshold control area [z-score] | 2.56 | ANOVA | 0.48 (1.26) | 0.77 (1.46) | 0.88 (1.63) | 0.323 | 1.000 | 1.14 |
| QST: Pressure pain threshold most painful area [z-score] | 0.00 | ANOVA | -0.12 (1.46) | 0.27 (1.61) | 0.94 (2.11) | 0.006 | 0.261 | 5.23 |
| QST: Thermal sensory limen most painful area [z-score] | 1.28 | ANOVA | -0.46 (0.84) | -0.76 (1.07) | -0.99 (1.37) | 0.040 | 1.000 | 3.29 |
| QST: Vibration detection threshold most painful area [z-score] | 1.28 | ANOVA | -0.28 (1.19) | -0.72 (1.57) | -1.8 (1.83) | 0.000 | 0.000* | 14.26 |
| QST: Warm detection threshold most painful area [z-score] | 1.28 | ANOVA | -0.03 (1.07) | -0.38 (1.4) | -0.53 (1.37) | 0.103 | 1.000 | 2.30 |
| QST: Wind-up ratio control area [z-score] | 2.56 | ANOVA | 0.07 (0.99) | 0.06 (1.11) | 0.03 (1.23) | 0.988 | 1.000 | 0.01 |
| QST: Wind-up ratio most painful area [z-score] | 0.00 | ANOVA | 0.13 (1.08) | -0.15 (1.6) | -0.1 (1.75) | 0.572 | 1.000 | 0.56 |
| Relationship status [single/in a committed relationship-married/separated-divorced/widowed] | 0.00 | Chi-Squared | 22/36/3/0 (36/59/5/0) | 14/22/5/1 (33/52/12/2) | 17/28/8/0 (32/53/15/0) | 0.887 | 1.000 | 0.24 |
| Sex [female/male] | 0.00 | Chi-Squared | 34/27 (56/44) | 24/18 (57/43) | 32/21 (60/40) | 0.947 | 1.000 | 0.11 |
| SSEP [pathological/physiological] | 8.97 | Chi-Squared | 4/53 (7/93) | 8/31 (21/79) | 8/38 (17/83) | 0.172 | 1.000 | 3.52 |
| SSR habituation control area [% change] | 14.10 | nt | nt | nt | nt | nt | nt | nt |
| SSR habituation most painful area [% change] | 10.90 | nt | nt | nt | nt | nt | nt | nt |
| Tiredness [no problems/slight problems/moderate problems/severe problems] | 0.00 | Chi-Squared | 34/26/1/0 (56/43/2/0) | 11/18/13/0 (26/43/31/0) | 4/18/19/12 (8/34/36/23) | 0.000 | 0.000* | 43.98 |
| TSP control area [% change] | 1.28 | ANOVA | 14.51 (24.67) | 11.38 (26.41) | 9.34 (27.15) | 0.569 | 1.000 | 0.57 |
| TSP most painful area [% change] | 7.05 | ANOVA | 8.42 (20.24) | 12.7 (18.48) | 14.71 (21.96) | 0.264 | 1.000 | 1.34 |

*Numeric data is shown as mean (standard deviation) and categorical data is shown as count (% of group). Abbreviations: CHEP: contact-heat evoked potential, CPM: conditioned pain modulation, CRPS: complex regional pain syndrome, HADS: hospital anxiety and depression scale, HC: healthy control, IPAQ-SF: international physical activity questionnaire – short form, LBP: low back pain, MAIA: multidimensional assessment of interoceptive awareness, nt: not tested (due to more than 10% missing data), PCS: pain catastrophizing scale, QST: quantitative sensory testing, SCI: spinal cord injury, SSEP: somatosensory evoked potential, SSR: sympathetic skin response, TSP: temporal summation of pain, *significant after adjustment*
